# Translation of the eHealth Impact Questionnaire for a Population of Dutch Electronic Health Users: Validation Study

**DOI:** 10.2196/13408

**Published:** 2019-08-26

**Authors:** Koen Ilja Neijenhuijs, Anja van der Hout, Evalien Veldhuijzen, Gwendolijne G M Scholten-Peeters, Cornelia F van Uden-Kraan, Pim Cuijpers, Irma M Verdonck-de Leeuw

**Affiliations:** 1 Department of Clinical, Neuro- and Developmental Psychology Amsterdam Public Health Research Institute Vrije Universiteit Amsterdam Amsterdam Netherlands; 2 Cancer Center Amsterdam Amsterdam UMC Amsterdam Netherlands; 3 Department of Otolaryngology-Head and Neck Surgery Amsterdam Public Health Research Institute Amsterdam UMC Amsterdam Netherlands; 4 Department of Human Movement Sciences Amsterdam Movement Sciences Vrije Universiteit Amsterdam Amsterdam Netherlands; 5 Department of Orthopedics and Research Kliniek ViaSana Mill Netherlands

**Keywords:** eHealth, evaluation, e-Health Impact Questionnaire, psychometrics

## Abstract

**Background:**

The eHealth Impact Questionnaire (eHIQ) provides a standardized method to measure attitudes of electronic health (eHealth) users toward eHealth. It has previously been validated in a population of eHealth users in the United Kingdom and consists of 2 parts and 5 subscales. Part 1 measures attitudes toward eHealth in general and consists of the subscales attitudes towards online health information (5 items) and attitudes towards sharing health experiences online (6 items). Part 2 measures the attitude toward a particular eHealth application and consists of the subscales confidence and identification (9 items), information and presentation (8 items), and understand and motivation (9 items).

**Objective:**

This study aimed to translate and validate the eHIQ in a Dutch population of eHealth users.

**Methods:**

The eHIQ was translated and validated in accordance with the COnsensus-based Standards for the selection of health status Measurement INstruments criteria. The validation comprised 3 study samples, with a total of 1287 participants. Structural validity was assessed using confirmatory factor analyses and exploratory factor analyses (EFAs; all 3 samples). Internal consistency was assessed using hierarchical omega (all 3 samples). Test-retest reliability was assessed after 2 weeks, using 2-way intraclass correlation coefficients (sample 1). Measurement error was assessed by calculating the smallest detectable change (sample 1). Convergent and divergent validity were assessed using correlations with the remaining measures (all 3 samples). A graded response model was fit, and item information curves were plotted to describe the information provided by items across item trait levels (all 3 samples).

**Results:**

The original factor structure showed a bad fit in all 3 study samples. EFAs showed a good fit for a modified factor structure in the first study sample. This factor structure was subsequently tested in samples 2 and 3 and showed acceptable to good fits. Internal consistency, test-retest reliability, convergent validity, and divergent validity were acceptable to good for both the original as the modified factor structure, except for test-retest reliability of one of the original subscales and the 2 derivative subscales in the modified factor structure. The graded response model showed that some items underperformed in both the original and modified factor structure.

**Conclusions:**

The Dutch version of the eHIQ (eHIQ-NL) shows a different factor structure compared with the original English version. Part 1 of the eHIQ-NL consists of 3 subscales: attitudes towards online health information (5 items), comfort with sharing health experiences online (3 items), and usefulness of sharing health experiences online (3 items). Part 2 of the eHIQ-NL consists of 3 subscales: motivation and confidence to act (10 items), information and presentation (13 items), and identification (3 items).

## Introduction

Currently, patients and care providers are encouraged to use electronic health (eHealth) apps to improve health care, including self-management [1,2]. A standardized measure to evaluate eHealth apps throughout the development process is needed. In the Netherlands, more than 98% of the population has access to the internet [3], and the use of eHealth apps is stimulated by both government and health care organizations. Internationally, the access to the internet is also growing rapidly. A standardized measure to evaluate eHealth apps is therefore much needed. However, evaluating eHealth apps is difficult because of a number of factors, including the difficulty of creating controlled experiments and confounding variables such as proficiency with the internet [4], and the continued development of eHealth app in comparison with more traditional forms of health care. Currently, evaluation of eHealth apps usually consists of 2 components: testing efficacy using randomized controlled trials (RCTs) and in-depth evaluation of the content of the app using structured and unstructured interviews. These methods require a large investment of time and resources. Given the rapid development of technology, this creates a state of *playing catch-up* for eHealth developers. A standardized way of evaluating eHealth apps can be invaluable in the process of constant development and evaluation. Although some such standardized measures exist (eg, the system usability, which measures the usability of software apps), they do not offer similar insight into the user experience through interviews.

In 2013, Kelly et al [5] developed the eHealth Impact Questionnaire (eHIQ) to measure the self-reported impact of eHealth on its users. On the basis of 5 themes, which were identified from interviews, the questionnaire consists of 2 parts. The first part (11 items) measures the overall attitude of eHealth users regarding eHealth, consisting of 2 subscales: *attitudes towards online health information* (5 items) and *attitudes towards sharing health experiences online* (6 items). The second part (26 items) measures the attitude of eHealth users regarding a specific eHealth app, consisting of 3 subscales: *confidence and identification* (9 items), *information and presentation* (8 items), and *understand and motivation* (9 items). This questionnaire was validated in 2015 for the British eHealth users [6].

The goal of this study was to translate and validate the eHIQ in a Dutch population of eHealth users—resulting in the Dutch version called eHIQ-NL—according to the COnsensus-based Standards for the selection of health status Measurement INstruments (COSMIN) criteria [7]. These criteria provide a systematic roadmap for appropriate analyses and interpretation of different types of validity and reliability. To our knowledge, the eHIQ has not been previously translated and/or validated outside of the original development and validation [5,6].

In the first study (the main study), Dutch users of the website Kanker.nl (an eHealth website for Dutch cancer patients) completed both parts of the eHIQ twice. In the second study, the first part of the eHIQ was completed by Dutch cancer survivors who were invited to participate in a survey on supportive cancer care, which was part of a randomized controlled trial to evaluate the efficacy of Oncokompas (an eHealth self-management app that supports Dutch cancer survivors in finding and obtaining optimal supportive care) [8]. In the third study, the second part of the eHIQ was completed by Dutch patients who had undergone orthopedic surgery and were participants in a pilot study of an app providing health information regarding pre- and postoperative care.

## Methods

### Translation

The questionnaire was translated from English into Dutch by 2 independent translators: 1 eHealth expert and 1 language expert who is a Dutch native and fluent in English. These translations were combined into a single Dutch questionnaire by 2 independent reviewers. In case of discrepancies, the final translation was decided by consensus. The Dutch translation was then translated back into English by 2 independent experts in language who are English natives and fluent in Dutch. The back-translated questionnaire was compared with the original English version by 2 independent reviewers. Discrepancies between the back-translated and the original English questionnaire were discussed, and some final changes were made. An example copy of the final translated questionnaire can be found in Multimedia Appendix 1.

### Recruitment and Procedure

Due to the results of the main study (study sample 1), the eHIQ was subsequently presented to 2 other samples of (prospective) eHealth users (study samples 2 and 3).

#### Study Sample 1

Dutch users of the national website Kanker.nl (an eHealth website for cancer patients) who had signed to participate in scientific research were asked to fill in both parts of the eHIQ-NL twice, with an interval of 2 weeks. On the second measurement, they were also asked to answer 2 questions designed to gauge attitudes to eHealth apps; 1 question asked them to grade their satisfaction with Kanker.nl on a 10-point scale (overall satisfaction), whereas the other question asked how likely they were to recommend Kanker.nl to a fellow cancer patient (the Net Promoter Score, NPS). They were further asked to fill in the 5-level EuroQol-5D version (EQ-5D-5L), which measures self-reported health-related quality of life [9].

#### Study Sample 2

A random sample of cancer survivors (breast cancer, colorectal cancer, head and neck cancer, or lymphoma) was drawn from the Netherlands Cancer Registry and invited to complete a survey on supportive cancer care, which was part of an RCT investigating the efficacy of Oncokompas (an eHealth self-management app that supports Dutch cancer survivors in finding and obtaining optimal supportive care) [8]. Patients were excluded who had severe cognitive impairment, insufficient mastery of the Dutch language, physical inability to complete a questionnaire, or received palliative care. Participants with internet access filled in the first part of the eHIQ-NL during the survey on supportive care. They were also asked to fill in the Functional, Communicative and Critical Health Literacy (FCCHL) scales (Cronbach α was .94 in the current sample), which measures the capacity of individuals to access, understand, and use health information [10], and the European Organisation for Research and Treatment of Cancer core quality of life questionnaire, version 3.0 Cronbach α was .98 in the current sample), which measures cancer-related quality of life [11]. Medical ethical approval was provided by the Medical Ethics Review Board of the VU Medical Center in Amsterdam, the Netherlands (reference number 2015.523).

#### Study Sample 3

Patients were recruited from a single clinic (ViaSana, Mill, The Netherlands) to participate in a pilot study of an app providing health information regarding pre- and postoperative care. Patients were eligible when aged older than 18 years and had undergone orthopedic surgery. Patients were excluded if they were not accessible by e-mail. Participants filled in the second part of the eHIQ-NL up to 2 weeks after using the app. Participants also filled in the System Usability Scale (SUS; Cronbach α was .90 in the current sample), which measures the usability of software apps [12], and 2 questions designed to gauge attitudes to eHealth apps, 1 question asked them to grade the app on a 10-point scale, whereas the other question asked how likely they were to recommend the app to a fellow patient. Medical ethical approval was provided by Medical Ethics Review Board of the Elisabeth Hospital in Tilburg, the Netherlands (reference number METC-T2012-11).

### Statistical Analysis

All analyses were performed in R version 3.3.3 [13]. Measurement properties were assessed in accordance with the COSMIN criteria [7].

#### Study Sample 1

First, structural validity was assessed with a combination of confirmatory factor analyses (CFAs) and exploratory factor analyses (EFAs). All CFAs were run using the cfa function of the lavaan package version 0.6-3 [14], whereas all EFAs were run using the efaUnrotate function of the semTools package version 0.5-1 [15], and Oblimin rotation was applied using the oblqRotate function of the semTools package version 0.5-1 [15].

Second, internal consistency was assessed by calculating hierarchical omega [16] using the reliability function of the semTools package version 0.5-1 [15]. Third, test-retest reliability was assessed by calculating a 2-way intraclass correlation coefficient (ICCs) between the 2 measurement times, using the icc function of the irr package version 0.84.1 [17]. Fourth, measurement error was assessed by calculating the standard error of measurement using the SE.Meas function of the psychometric package version 2.2 [18]. The smallest detectable change (SDC) was calculated by hand using the standard error of measurement.

Fifth, convergent validity and divergent validity were tested by correlating the subscales of the eHIQ-NL with the questions concerning satisfaction with Kanker.nl and the NPS (a positive correlation was hypothesized) and the EQ-5D-5L of which the items for *daily activities* and *anxiety or depression* were assumed to show a positive correlation. No correlation was hypothesized to exist between the eHIQ-NL and the remaining EQ-5D-5L items. Correlations were calculated using the rcorr function of the Hmisc package [19].

Sixth and last, a graded response model was fit using the grm function of the ltm package [20]. Item information curves were plotted for each subscale to describe the information provided by items across the item trait level (ie, the construct measured by the subscale).

#### Study Sample 2

Structural validity was assessed with a combination of CFAs and EFAs. Internal consistency was assessed with hierarchical omega. Divergent validity was tested by correlating the subscales of the eHIQ-NL with the FCCHL, as no correlation was hypothesized to exist. Finally, a graded response model was fit. All analyses were performed using the same functions and R packages as in study sample 1.

#### Study Sample 3

Structural validity was assessed with a combination of CFAs and EFAs. Internal consistency was assessed with hierarchical omega. Convergent validity was tested by correlating the subscales of the eHIQ-NL with the SUS and the questions concerning the grade and likelihood of recommending the app, as positive correlations were hypothesized. Finally, a graded response model was fit. All analyses were performed using the same functions and R packages as in study sample 1.

## Results

### Study Population

Table 1 shows the demographic and clinical characteristics of the 3 study samples. In study sample 1, 304 cancer survivors participated with a mean age of 58.12 years (SD=11.26), and 177 were female (58.2%, 177/304). The study sample consisted of more than 17 cancer diagnoses; most were diagnosed with breast cancer (27.1%, 82/340) or prostate cancer (13.8%, 42/340). The feasibility of the eHIQ-NL was good: of the 304 participants who started the first measurement, 288 (94.7%, 288/304) completed the eHIQ-NL. A total of 242 (79.6%, 242/304) participants started the second measurement, of which 217 (71.4%, 217/304) completed all questionnaires.

In study sample 2, 566 cancer survivors completed the first part of the eHIQ-NL with a mean age of 64.18 years (SD=10.65), and 351 (62.1%, 351/565) were female. The study sample consisted of 4 cancer diagnoses: breast cancer (39.2%, 222/566), colorectal cancer (29.7%, 168/566), head and neck cancer (19.1%, 108/566), and lymphoma (12.0%, 68/566).

In study sample 3526 orthopedic patients completed the second part of the eHIQ-NL with a median age of 59.00 years (interquartile range=50-66), and 267 were female (50.7%, 267/526). The study sample consisted of patients who underwent various orthopedic surgeries; the main group had undergone a total knee arthroplasty (31.1%, 164/526).

**Table 1:** Descriptive statistics of the study population.

Study sample, characteristic		Study sample 1 (N=288)	Study sample 2 (N=566)	Study sample 3 (N=526)
Age, mean (SD)		58.12 (11.26)	64.18 (10.65)	59.00 (50-66)^a^
**Gender, n (%)**				
	Male	126	214	259
	Female	177	351	267
**Diagnosis, n (%)**				
	Breast cancer	82 (26.9)	222 (39.2)	—^b^
	Miscellaneous cancer	47 (15.4)	—	—
	Prostate cancer	42 (13.8)	—	—
	Lymphoma	20 (6.5)	68 (12.0)	—
	Colon cancer	18 (5.9)	—	—
	Skin cancer	18 (5.9)	—	—
	Lung cancer	17 (5.5)	—	—
	Bladder and kidney cancer	17 (5.5)	—	—
	Rectal cancer	14 (4.6)	—	—
	Head and neck cancer	12 (3.9)	108 (19.1)	—
	Esophageal cancer	10 (3.2)	—	—
	Leukemia	10 (3.2)	—	—
	Other	33 (8.8)	—	—
	Colorectal cancer	—	168 (29.7)	—
	Total knee arthroplasty	—	—	164 (31.2)
	Total hip arthroplasty	—	—	89 (16.9)
	Anterior cruciate ligament reconstruction	—	—	56 (10.6)
	Knee arthroscopy	—	—	47 (8.9)
	Cuff repair	—	—	30 (5.7)
	High tibial osteotomy	—	—	23 (4.4)
	Lumbar discectomy	—	—	17 (3.2)
	Acromionplasty	—	—	14 (2.7)
	Remaining group	—	—	86 (16.3)

^a^Median (interquartile range).

^b^Not assessed in this study.

^c^Remaining group: shoulder arthroplasty, femoral osteotomy, patella stabilization (medial patellofemeral ligament), mortons neurom, hallux valgus/rigidus, exostosis, and talocrual arthrodesis.

### Study Sample 1

#### Structural Validity

A CFA was run on a 2-level hierarchical model, with the specified subscales as first-order factors, and the 2 different sections (general attitude and specific attitude) as second-order factors. This model had a bad fit (minimum discrepancy per degree of freedom [CMIN]=2.61, adjusted goodness-of-fit index [AGFI]=0.719, Comparative Fit Index [CFI]=0.752, Tucker-Lewis index [TLI]=0.753, standardized root mean square residual [SRMR]=0.076, and root mean square error of approximation [RMSEA]=0.075 [0.070-0.079]). Inspecting the modification indices revealed cross-loadings of items on the second-order factors. Such cross-loadings made sense when looking at the content of the items (eg, items on information on the specific eHealth tool showing cross-loadings with general attitude toward health information); however, shifting items from one section to another made no theoretical or practical sense. Therefore, 2 CFAs were run separately for each section, removing the second-order factor from the analysis.

The fit for the first part of the questionnaire was better than the first model fit, but not yet acceptable (CMIN=5.14, AGFI=0.796, CFI=0.847, TLI=0.804, SRMR=0.074, and RMSEA=0.118 [0.103-0.134]). A 3-factor EFA using Oblimin rotation was run to investigate an alternative to the original factor structure. This model showed a good fit (CMIN=3.16, AGFI=0.989, CFI=0.954, TLI=0.898, SRMR=0.032, and RMSEA=0.085 [0.065-0.107]). The 3 factors were interpretable (Multimedia Appendix 2), with the subscale *attitudes towards sharing health experiences online* being split into the 2 factors *comfort with sharing health experiences online* and *usefulness of sharing health experiences online*. The third factor was identical to the original factor of *attitudes towards online health information*.

The fit for the second part of the questionnaire was also better than the first model fit, but not yet acceptable (CMIN=3.20, AGFI=0.747, CFI=0.755, TLI=0.731, SRMR=0.082, and RMSEA=0.087 [0.081-0.094]). A 4-factor EFA using Oblimin rotation was run to investigate an alternative to the original factor structure. The model showed a good fit (CMIN=2.01, AGFI=0.988, CFI=0.914, TLI=0.876, SRMR=0.037, and RMSEA=0.059 [0.051-0.067]), but the factor structure was not clearly interpretable, many items had double loadings, and the fourth factor had very low factor loadings. A 5-factor EFA using Oblimin rotation showed a similar fit (CMIN=1.93, AGFI=0.988, CFI=0.928, TLI=0.886, SRMR=0.033, and RMSEA=0.057 [0.048-0.065]). Although the double loadings were mostly taken care of, the loadings on the fourth and fifth factors were very low.

A 3-factor EFA using Oblimin rotation was run to investigate problematic items. Items 10, 8, 16, 4, 17, and 11 showed double loadings and no clear distinction to any one factor. Removing these items and performing a CFA on the original factor structure resulted in a bad fit (CMIN=3.39, AGFI=0.779, CFI=0.786, TLI=0.757, SRMR=0.084, and RMSEA=0.091 [0.083-0.099]). Running an EFA using Oblimin rotation on the same subset of items resulted in a good fit (CMIN=2.22, AGFI=0.990, CFI=0.920, TLI=0.886, SRMR=0.041, and RMSEA=0.062 [0.052-0.072]), but with a different factor structure than theorized (Multimedia Appendix 2): the first factor being a combination of items from the subscales *confidence and identification* and *Understanding and motivation* and interpretable as *motivation and confidence to act*; the second factor being identical to the original subscale *information and presentation* with the addition of item 2; and the third factor consisting of 3 items from the subscale *confidence and identification* and interpretable as *identification*.

#### Internal Consistency

Multimedia Appendix 3 shows the results on internal consistency of the original factor structure and the modified factor structure, respectively. All values were acceptable (omega>0.70), and the values of the original first part and the modified first part were comparable. The values of the modified second part were better than of the original second part.

#### Test-Retest Reliability

Table 2 shows the results on test-retest reliability of the original factor structure and the modified factor structure. All original subscales, except for *attitudes towards sharing health experiences online* (ICC=0.63) showed acceptable ICCs (ICC>0.70). All modified subscales, except for *comfort with sharing health experiences online* (ICC=0.62) and *usefulness of sharing health experiences online* (ICC=0.53) showed acceptable ICCs (ICC>0.70). The ICCs for the original factor structure and the modified factor structure were comparable.

**Table 2:** Test-retest reliability.

Structure, subscale		ICC^a^	CI
**Original factor structure**			
	Attitudes towards online health information	0.71	0.64-0.77
	Attitudes towards sharing health experiences online	0.63	0.54-0.7
	Confidence and identification	0.73	0.66-0.78
	Information and presentation	0.72	0.64-0.78
	Understanding and motivation	0.74	0.67-0.8
**Modified factor structure**			
	Attitudes towards online health information	0.71	0.64-0.77
	Comfort with sharing health experiences online	0.62	0.53-0.69
	Usefulness of sharing health experiences online	0.53	0.43-0.62
	Motivation and confidence to act	0.76	0.7-0.81
	Information and presentation	0.73	0.66-0.79

^a^ICC: intraclass correation coefficient.

#### Measurement Error

Table 3 shows the results of the measurement error of the original factor structure and the modified factor structure. For the original factor structure, the SDC ranged between 15.77 and 26.18, which represents a measurement error of 15%-26% of the 100 subscale range. Consequently, we can be 95% certain that a change score larger than 15% to 26% of the subscale range is not an artifact of measurement error. For the modified factor structure, the SDC ranged between 15.05 and 34.81, which represents a measurement error of 15% to 35% of the 100 subscale range. The highest SDCs were reported for the Part 1 *attitudes towards sharing health experiences online* (34.81) and *comfort with sharing health experiences online* (28.91) subscales. This makes sense, as both subscales only consisted of 3 items, and small scales are susceptible to high measurement error.

#### Convergent and Divergent Validity

All subscales correlated significantly with both the overall satisfaction and the NPS. The correlations between the subscales of the first part of the eHIQ-NL and the overall satisfaction and the NPS were small (*r*<0.30). There were either no significant or very small (*r*<0.20) correlations with the EQ-5D questions on daily activities and anxiety and depression. The 3 remaining EQ-5D items did not correlate significantly with any of the eHIQ-NL subscales (Table 4)

#### Graded Response Model

**Figure 1:**
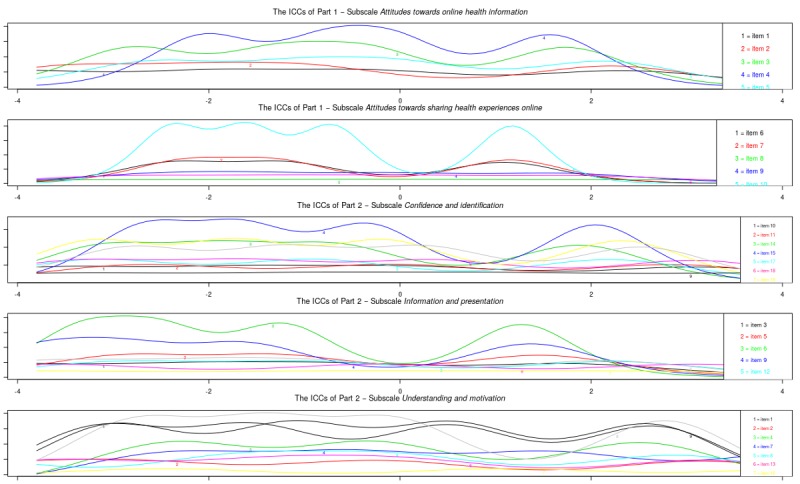
Study sample 1: item information curves (IICs) of original subscales.

Figure 1 shows the item information curves for the original subscales. A number of items of part 1 did not provide much extra information to the subscale: items 1, 2, 8, 9, and 11. Notably, most items in the subscale attitudes *towards sharing health experiences online* provided information at the same item trait levels. A number of items of Part 2 also did not provide much extra information to the subscale: items 2, 10, 11, 13, 16, 23, and 25. Notably, items 10, 11, and 16 were items that fit poorly in the factor analysis.

**Figure 2:**
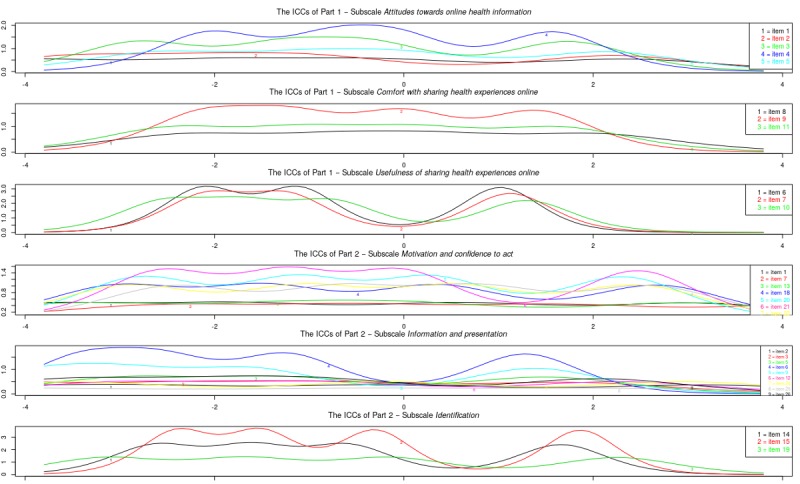
Study sample 1: item information curves (IICs) of the modified subscales.

Figure 2 shows the item information curves of the modified subscales. Of part 1, the information of the subscale *comfort with sharing health experiences online* was rather low across the entire latent trait spectrum. For the subscale *usefulness of sharing health experiences online* information was high on certain points of the latent trait spectrum, but all 3 items overlap almost completely. Of part 2, the subscale *motivation and confidence to act* showed a good range of information across latent trait levels. However, 3 items hardly contributed information (items 1, 7, and 13). The subscale *information and presentation* still suffered from multiple items adding little information, as well as a lot of overlap. Finally, the subscale *identification* showed a good range of information as well as high peaks for all 3 items, but still a lot of overlap between items on information range.

**Table 3:** Measurement error.

Structure, subscale Original factor structure		SEM^a^	SDC^b^
**Original factor structure**			
	Attitudes towards online health information	9.14	25.32
	Attitudes towards sharing health experiences online	9.44	26.18
	Confidence and identification	6.79	18.83
	Information and presentation	5.69	15.77
	Understanding and motivation	6.33	17.54
**Modified factor structure**			
	Attitudes towards online health information	9.14	25.32
	Comfort with sharing health experiences online	12.56	34.81
	Usefulness of sharing health experiences online	10.43	28.90
	Motivation and confidence to act	7.19	19.93
	Information and presentation	5.43	15.05

^a^SEM: standard error of measurement.

^b^SDC: smallest detectable change.

**Table 4:** Convergent and divergent validity.

Study sample		Original factor structure					Modified factor structure					
		OHI^a^	SHEO^b^	C&I^c^	I&P^d^	U&M^e^	OHI	CSHEO^f^	USHEO^g^	M&CA^h^	I&P	ID^i^
**A: Study sample 1—convergent**												
	Grade	0.17^j^	0.28^k^	0.50^k^	0.47^k^	0.49^k^	0.17^j^	0.23^k^	0.27^k^	0.46^k^	0.49^k^	0.35^k^
	NPS^l^	0.21^m^	0.25^k^	0.48^k^	0.44^k^	0.44^k^	0.21^m^	0.19^m^	0.26^k^	0.43^k^	0.45^k^	0.37^k^
	EQ-5D^n^—daily activities	−0.01	0.07	−0.05	0.00	−0.05	−0.01	0.08	0.04	−0.05	0.00	0.01
	EQ-5D—anxiety/depression	−0.07	0.00	−0.13	−0.18^m^	−0.14^j^	−0.07	0.06	−0.07	−0.15^j^	−0.18^m^	−0.02
**B: Study sample 1—divergent**												
	EQ-5D—mobility	−0.04	0.02	−0.04	−0.05	−0.02	−0.04	0.01	0.02	−0.01	−0.03	0.01
	EQ-5D—selfcare	0.02	0.07	−0.04	−0.03	0.03	0.02	0.06	0.07	0.01	−0.02	−0.04
	EQ-5D—pain	−0.04	0.00	−0.08	−0.06	−0.08	−0.04	0.03	−0.04	−0.09	−0.06	−0.03
**C: Study sample 2—divergent**												
	FCCHL^o^	0.14^m^	0.03	—^p^	—	—	0.14^m^	−0.01	0.06	—	—	—
	EORTC QLQ-C30^q^	0.01	0.01	—	—	—	0.01	0.00	0.02	—	—	—
**D: Study sample 3—convergent**												
	System Usability Scale	—	—	0.29^k^	0.53^k^	0.39^k^	—	—	—	0.30^k^	0.55^k^	0.12^m^
	NPS	—	—	0.46^k^	0.42^k^	0.52^k^	—	—	—	0.48^k^	0.45^k^	0.31^k^
	Grade^r^			0.59^k^	0.53^k^	0.62^k^				0.58^k^	0.55^k^	0.43^k^

^a^OHI: attitudes towards online health information.

^b^SHEO: attitudes towards sharing health experiences online.

^c^C&I: confidence and identification.

^d^I&P: information and presentation.

^e^U&M: understanding and motivation.

^f^CSHEO: comfort with sharing health experiences online.

^g^USHEO: usefulness of sharing health experiences online.

^h^M&CA: motivation and confidence to act.

^i^ID: identification.

^j^*P*<.05.

^k^*P*<.01.

^l^NPS: Net Promoter Score.

^m^*P*<.001.

^n^EQ-5D: EuroQol-5D.

^o^FCCHL: Functional, Communicative and Critical Health Literacy scale.

^p^Not applicable.

^q^EORTC QLQ-C30: European Organisation for Research and Treatment of Cancer core quality of life questionnaire, version 3.0.

^r^Grade: overall satisfaction.

### Study Sample 2

#### Structural Validity

A CFA was run with the 2 original subscales as first-order factors. This model had a bad fit (CMIN=8.13, AGFI=0.829, CFI=0.893, TLI=0.863, SRMR=0.054, and RMSEA=0.113 [0.102-0.124]). A second CFA was run with the factor structure found in study 1. This model had a barely acceptable fit (CMIN=7.37, AGFI=0.849, CFI=0.909, TLI=0.878, SRMR=0.049, and RMSEA=0.107 [0.096-0.118]). An EFA using Oblimin rotation was run with 3 factors to determine possible deviations from the 3 subscales found in study 1. This model had a good fit (CMIN=4.86, AGFI=0.978, CFI=0.966, TLI=0.926, SRMR=0.025, and RMSEA=0.084 [0.069-0.098]). The 3 factors (Multimedia Appendix 2) were identical to the subscales found in study 1, except for item 8 loading on both subscales concerning the sharing of health experiences online.

#### Internal Consistency

Multimedia Appendix 3 shows the internal consistency of the original factor structure and the modified factor structure. All values were acceptable (omega>0.70) and comparable between both factor structures.

#### Divergent Validity

For both the original and the modified factor structure, only the subscale *attitudes toward online health information* showed a significant correlation with the FCCHL (Table 4). However, this correlation is small enough to be acceptable for divergent validity (*r*<0.15).

#### Graded Response Model

**Figure 3:**
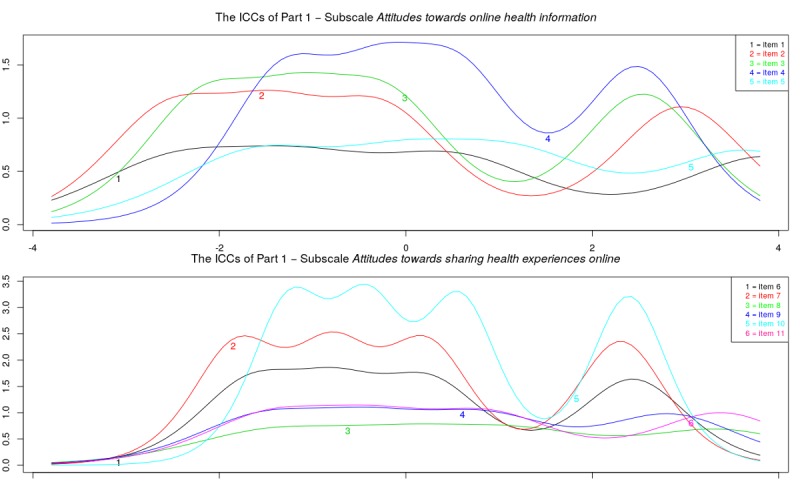
Study sample 2: item information curves (IICs) of original subscales.

**Figure 4:**
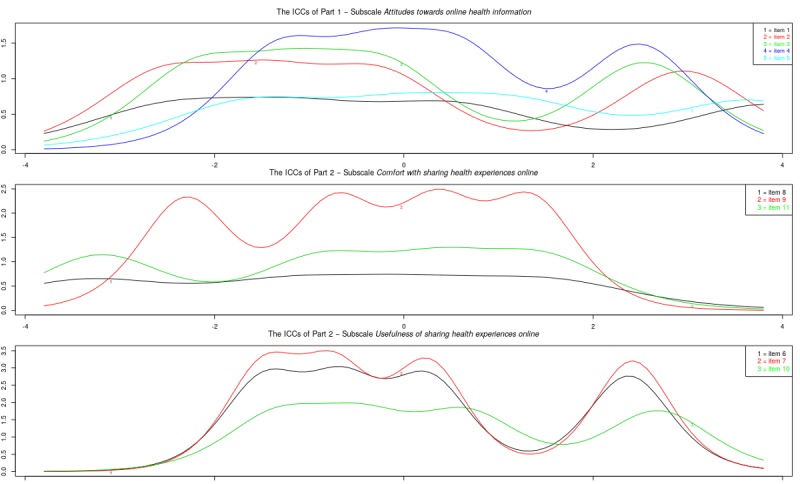
Study sample 2: item information curves (IICs) of modified subscales.

Figure 3 shows the item information curves for the original subscales of part 1. A number of items do not provide much extra information over the others: items 5, 8, and 9. Figure 4 shows the item information curves of the modified subscales of part 1. The information of the subscale *comfort with sharing health experiences online* showed large dips on certain levels of ability. For the subscale *usefulness of sharing health experiences online* information was high on certain points of the latent trait spectrum, but the items overlap a great deal.

### Study Sample 3

#### Structural Validity

A CFA was run with the 3 original subscales as first-order factors. This model had a slightly below acceptable fit (CMIN=5.568, AGFI=0.717, CFI=0.811, TLI=0.792, SRMR=0.092, and RMSEA=0.093 [0.089-0.098]). A second CFA was run with the 3 subscales found in study sample 1. This model had an acceptable fit (CMIN=4.447, AGFI=0.828, CFI=0.889, TLI=0.873, SRMR=0.075, and RMSEA=0.081 [0.075-0.087]). An EFA using Oblimin rotation was run with 3 factors and including the items that were deemed problematic in study sample 1 to determine whether including them would result in a better fit. This model had a good fit (CMIN=2.496, AGFI=0.990, CFI=0.948, TLI=0.932, SRMR=0.029, and RMSEA=0.053 [0.048-0.059]).

In the EFA, items 8, 11, and 17 showed no problematic cross-loadings. Items 4, 10, and 16 did show problematic cross-loadings, but not as extreme as in study sample 1 (Multimedia Appendix 2). Items 8 and 10 were found to load most highly on the factor representing *motivation and confidence to act*. Items 4, 11, 16, and 17 were found to load most highly on the factor representing *information and presentation*. Beyond the problematic items, only 1 item loaded differently than in study sample 1: item 20 loaded as highly on the factor representing *motivation and confidence to act* (on which it loaded in study sample 1) as it did on the factor representing *identification*.

#### Internal Consistency

Multimedia Appendix 3 shows the internal consistency of the original factor structure, the modified factor structure without previously problematic items, and the modified factor structure with previously problematic items, respectively. The internal consistency of the modified factor structure with previously problematic items is represented by Cronbach alpha instead of Omega, as Omega is based on factor variance and unsuitable for factor structures fit based on EFAs. All values, except for the original subscale *information and presentation* (omega=0.65), were acceptable and comparable between the 3-factor structures.

#### Convergent Validity

Both the original and modified subscales correlated significantly with the SUS, NPS, and *grade* questions (Table 4). All correlations were acceptable for convergent validity (*r*>0.30), except for the original subscale *confidence and identification* with the SUS (*r*=0.29) and the modified subscale *identification* with the SUS (*r*=0.12).

#### Graded Response Model

**Figure 5:**
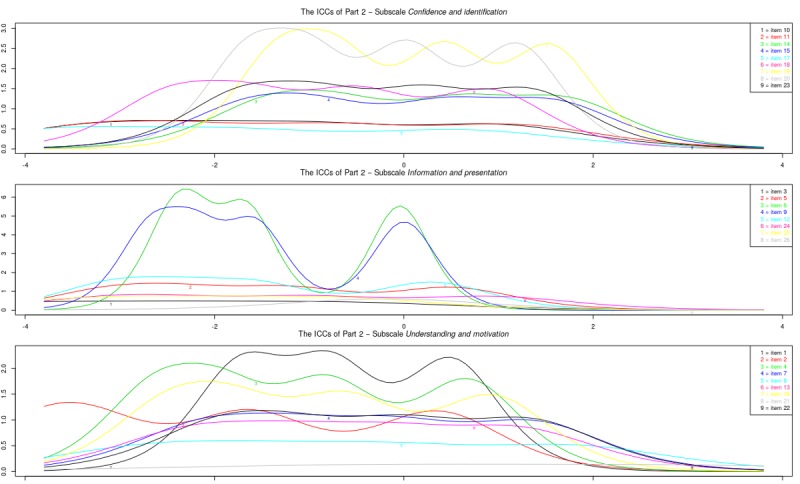
Study sample 3: item information curves (IICs) of original subscales.

**Figure 6:**
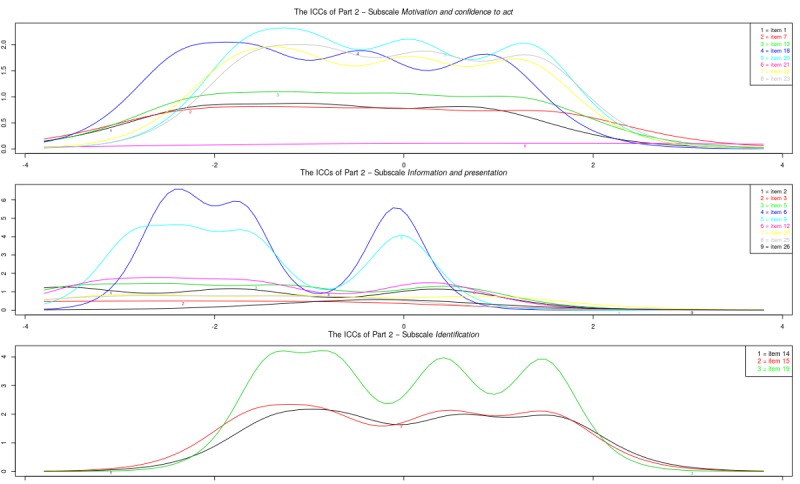
Study sample 3: item information curves (IICs) of modified subscales.

Figure 5 shows the item information curves for the original subscales of part 2. With a large number of items per scale, there was a good range of information across latent trait levels. Some items did not add much to the information provided by other items: items 3, 8, 10, 11, 17, 21, 24, 25, and 26. Notably, items 8, 10, 11, and 17 were items that were judged problematic in study 1. Figure 6 shows the item information curves of the modified subscales of part 2. The subscale *motivation and confidence to act* showed a good range of information across latent trait levels. However, 3 items hardly contributed information: items 1, 7, and 13. The subscale *information and presentation* still suffered from multiple items adding little information, as well as a lot of overlap. Finally, the subscale *identification* showed a good range of information as well as high peaks for all 3 items, but still a lot of overlap.

## Discussion

### Principal Findings

In this study, the eHIQ was translated into Dutch, and the measurement properties were investigated. Feasibility was good: more than 94% of participants in the main study completed the eHIQ-NL. The eHIQ-NL showed a different factor structure compared with the original English version. Part 1 of the eHIQ-NL consists of 3 subscales: *attitudes towards online health information* (5 items), *comfort with sharing health experiences online* (3 items), and *usefulness of sharing health experiences online* (3 items). Part 2 of the eHIQ-NL consists of 3 subscales: *motivation and confidence to act* (10 items), *information and presentation* (13 items), and *identification* (3 items). These factor structures were replicated in subsequent samples and altogether showed acceptable to good internal consistency, test-retest reliability, and construct validity.

### Limitations

Limitations of this study are some underperforming measurement properties of the modified factor structure. In particular, test-retest reliability for *comfort with sharing health experiences online* and *usefulness of sharing health experiences online* (ICC=0.62 and ICC=0.53, respectively) was below acceptable threshold. Notably, the original subscale comprised these 2 subscales *attitudes towards sharing health experiences online* also underperformed on test-retest reliability (ICC=0.63).

Furthermore, the correlations testing convergent validity were small in the main study (*r*<0.30), as well as some smaller correlations in study sample 3 for the subscales *confidence and identification* (*r*=0.29), and the modified subscale *identification* (*r*=0.12). We recognize that this may be because of subpar a priori hypotheses in regard to the EQ-5D (study 1) and the SUS (study 3). The reasoning for these hypotheses was somewhat tenuous. For the first sample, we expected the specific eHealth app Kanker.nl to provide useful information for patients with issues regarding daily activities and anxiety/depression resulting in a correlation between a higher score on these issues and eHIQ scores. For the third sample, we expected a higher usability score to be correlated to higher eHIQ scores, but we recognize that the subscales *confidence and identification* and *identification* may be theoretically unrelated to usability. Further research is necessary to further investigate test-retest reliability and construct validity of the eHIQ-NL. Future validations in different nationalities and different patient populations may shed more light on these measurement properties.

### Comparison With Prior Work

The findings of this study do not entirely match the findings of the original validation of the eHIQ for the British population [6]. The differences may be the results of a number of differences between the current and previous validation studies. The first explanation is that in the translation of the questionnaire, the meaning of some items may have changed. Although we followed a strict protocol for the translation, this explanation cannot be ruled out.

The second explanation can be found in the use of a different study populations. The original validation study presented the eHIQ to a range of health groups, who were not necessarily eHealth users at the time of the study. The participants in the original validation study were invited to the laboratory and were briefly (at least 15 min) acquainted with an eHealth app relevant to their personal health situation [6]. This study presented the eHIQ-NL to eHealth users who were familiar with the app under investigation (study samples 1 and 3) and noncurrent eHealth users. Furthermore, the current validation study presented the eHIQ-NL only to cancer patients (study samples 1 and 2) and patients with musculoskeletal disorders (study sample 3). As such, the populations differ quite a bit beyond nationality.

We realize that the results of this study present complexities to which subscales should be adhered to in case the user of the eHIQ-NL aims to compare their results with international samples. In such cases, we recommend that, besides the results using the subscales as presented in this study, the results using the original subscales are also reported. The caveat is that one cannot be sure of the structural validity using this method, and we recommend factor analysis to back up any such interpretation.

### Conclusions

Nevertheless the limitations specified above, the eHIQ-NL shows a consistent factor structure, sufficient internal consistency, and mostly sufficient test-retest reliability and construct validity. The eHIQ-NL is a valid and reliable tool for measuring attitudes of eHealth users. Interested users can contact Oxford Innovations (healthoutcomes@innovation.ox.ac.uk) for a license to use the eHIQ.

## Appendix

Multimedia Appendix 1 Example copy of Dutch version of the eHealth Impact Questionnaire.

Multimedia Appendix 2 Structural validity: exploratory factor analysis factor loadings.

Multimedia Appendix 3 Internal consistency.

## Abbreviations

AGFI: adjusted goodness-of-fit index
CFA: confirmatory factor analyses
CFI: Comparative Fit Index
CMIN: minimum discrepancy per degree of freedom
COSMIN: Consensus-based Standards for the selection of health status Measurement INstruments
EFA: exploratory factor analyses
eHealth: electronic health
eHIQ: eHealth Impact Questionnaire
eHIQ-NL: Dutch version of the eHealth Impact Questionnaire
EQ-5D-5L: 5-level EuroQol-5D version
FCCHL: Functional, Communicative and Critical Health Literacy
ICC: intraclass correlation coefficient
NPS: Net Promoter Score
RCT: randomized controlled trialRMSEA: root mean square error of approximation
SDC: smallest detectable change
SRMR: standardized root mean square residual
SUS: System Usability Scale
TLI: Tucker-Lewis Index
