# Supplementary material for: Translation of the eHealth Impact Questionnaire for a Population of Dutch Electronic Health Users: Validation Study
Source: J Med Internet Res. 2019 Aug 26;21(8):e13408. doi: 10.2196/13408 (PMC6732971; doi:10.2196/13408)
Supplement: Multimedia Appendix 1 [file jmir_v21i8e13408_app1.pdf]

De e – Health Impact Vragenlijst

Deel 1

In dit onderdeel wordt gevraagd naar ***uw** algemene houding tegenover gezondheidsgerelateerde websites*.

In dit gedeelte kan *'gezondheidsgerelateerde websites'* staan voor websites die feitelijke gezondheidsinformatie bevatten, ervaringsverhalen over gezondheid van anderen, blogs over gezondheid of discussieforums over gezondheid.

Start alstublieft met het beantwoorden van de onderstaande vragen.

© 2013, Oxford University Innovation Limited. Alle rechten voorbehouden.

De auteurs, prof. dr. Crispin Jenkinson, mw. Laura Kelly en prof. dr. Sue Ziebland, hebben aanspraak gemaakt op hun morele rechten.

	Selecteer het vakje dat op u van toepassing is.				
**In hoeverre bent u het wel of niet eens met de volgende uitspraken?**	Helemaal mee oneens	Mee oneens	Noch mee eens, noch mee oneens	Mee eens	Helemaal mee eens
1. Het internet is een betrouwbare bron om mij te helpen begrijpen wat een arts mij vertelt.					
2. Het internet kan mensen helpen om te weten hoe het is om te leven met een gezondheidsprobleem.					
3. Het internet kan nuttig zijn om mensen te helpen beslissen of hun symptomen belangrijk genoeg zijn om een arts te raadplegen.					
4. Ik zou het internet gebruiken als ik hulp nodig zou hebben bij het maken van een beslissing over mijn gezondheid (bijvoorbeeld of ik een arts zou moeten raadplegen, medicatie zou moeten innemen of andere typen behandelingen zou moeten zoeken).					

Voor vragen gerelateerd aan deze vragenlijst, neem alstublieft contact op met: XXXX

Gaat u verder op de volgende pagina

© 2013, Oxford University Innovation Limited. Alle rechten voorbehouden.

De auteurs, prof. dr. Crispin Jenkinson, mw. Laura Kelly en prof. dr. Sue Ziebland, hebben aanspraak gemaakt op hun morele rechten.

	Selecteer het vakje dat op u van toepassing is.				
**In hoeverre bent u het wel of niet eens met de volgende uitspraken?**	Helemaal mee oneens	Mee oneens	Noch mee eens, noch mee oneens	Mee eens	Helemaal mee eens
5. Ik zou het internet gebruiken om na te gaan of de arts mij passend advies geeft.					
6. Het internet is een goede manier om andere mensen te vinden die vergelijkbare gezondheidsproblemen ervaren.					
7. Het kan behulpzaam zijn om gezondheids-gerelateerde ervaringen van andere mensen op het internet te zien.					
8. Het internet is nuttig als je niet wilt vertellen aan mensen in je omgeving (bijvoorbeeld uw familie of collega's) hoe je je voelt.					

Voor vragen gerelateerd aan deze vragenlijst, neem alstublieft contact op met: XXXX

Gaat u verder op de volgende pagina

© 2013, Oxford University Innovation Limited. Alle rechten voorbehouden.

De auteurs, prof. dr. Crispin Jenkinson, mw. Laura Kelly en prof. dr. Sue Ziebland, hebben aanspraak gemaakt op hun morele rechten.

	Selecteer het vakje dat op u van toepassing is.				
**In hoeverre bent u het wel of niet eens met de volgende uitspraken?**	Helemaal mee oneens	Mee oneens	Noch mee eens, noch mee oneens	Mee eens	Helemaal mee eens
9. Het kan geruststellend zijn om te weten dat ik op elk moment van de dag of nacht terecht kan op gezondheidsgerelateerde websites.					
10. Het internet is een goede manier om andere mensen te vinden die geconfronteerd zijn met gezondheidsgerelateerde beslissingen waar ik mogelijk ook mee wordt geconfronteerd.					
11. Het bekijken van websites over gezondheid stelt me gerust dat ik niet alleen ben met mijn gezondheidszorgen.					

Voor vragen gerelateerd aan deze vragenlijst, neem alstublieft contact op met: XXXX

Gaat u verder op de volgende pagina

© 2013, Oxford University Innovation Limited. Alle rechten voorbehouden.

De auteurs, prof. dr. Crispin Jenkinson, mw. Laura Kelly en prof. dr. Sue Ziebland, hebben aanspraak gemaakt op hun morele rechten.

Volg alstublieft de onderstaande instructies op:

1. Klik op de onderstaande link naar de gezondheidsgerelateerde website. Er zal **een nieuwe pagina in uw browser openen**.

2. Neem 10 tot 15 minuten de tijd om naar de onderdelen van de website te surfen die **uw interesse hebben**.

3. Wanneer u klaar bent met surfen op de website, **keer dan terug naar deze pagina en klik op 'doorgaan'** om de resterende vragen te beantwoorden.

Gezondheidsgerelateerde website: [Voeg website in]

(Houdt u er rekening mee dat als u niet binnen 30 minuten naar deze vragenlijst terugkeert uw sessie zal verlopen)

Voor vragen gerelateerd aan deze vragenlijst, neem alstublieft contact op met: XXXX

Gaat u verder op de volgende pagina

© 2013, Oxford University Innovation Limited. Alle rechten voorbehouden.

De auteurs, prof. dr. Crispin Jenkinson, mw. Laura Kelly en prof. dr. Sue Ziebland, hebben aanspraak gemaakt op hun morele rechten.

Deel 2

In dit onderdeel wordt gevraagd naar ***uw mening** over de gezondheids-gerelateerde website die u zojuist heeft bekeken.*

	Selecteer het vakje dat op u van toepassing is.				
**Denkend aan de website die u net bekeken heeft, in hoeverre bent u het wel of niet eens met de volgende uitspraken?**	Helemaal mee oneens	Mee oneens	Noch mee eens, noch mee oneens	Mee eens	Helemaal mee eens
1. De website moedigt mij aan om acties te ondernemen die gunstig kunnen zijn voor mijn gezondheid.					
2. De website heeft een positieve kijk.					
3. De informatie op de website liet een gevoel van verwarring bij me achter.					
4. De website bevat nuttige tips over hoe het leven beter te maken.					
5. De website biedt een breed scala aan informatie.					

Voor vragen gerelateerd aan deze vragenlijst, neem alstublieft contact op met: XXXX

Gaat u verder op de volgende pagina

© 2013, Oxford University Innovation Limited. Alle rechten voorbehouden.

De auteurs, prof. dr. Crispin Jenkinson, mw. Laura Kelly en prof. dr. Sue Ziebland, hebben aanspraak gemaakt op hun morele rechten.

	Selecteer het vakje dat op u van toepassing is.				
**Denkend aan de website die u net bekeken heeft, in hoeverre bent u het wel of niet eens met de volgende uitspraken?**	Helemaal mee oneens	Mee oneens	Noch mee eens, noch mee oneens	Mee eens	Helemaal mee eens
6. De taal op de website maakte het gemakkelijk te begrijpen.					
7. Ik voel me meer geneigd om op mezelf te letten na het bezoeken van de website.					
8. Ik heb iets nieuws geleerd van de website.					
9. Ik kan de informatie op de website gemakkelijk begrijpen.					
10. De website bereidt me voor op wat er mogelijk gaat gebeuren met mijn gezondheid.					

Voor vragen gerelateerd aan deze vragenlijst, neem alstublieft contact op met: XXXX

Gaat u verder op de volgende pagina

© 2013, Oxford University Innovation Limited. Alle rechten voorbehouden.

De auteurs, prof. dr. Crispin Jenkinson, mw. Laura Kelly en prof. dr. Sue Ziebland, hebben aanspraak gemaakt op hun morele rechten.

	Selecteer het vakje dat op u van toepassing is.				
**Denkend aan de website die u net bekeken heeft, in hoeverre bent u het wel of niet eens met de volgende uitspraken?**	Helemaal mee oneens	Mee oneens	Noch mee eens, noch mee oneens	Mee eens	Helemaal mee eens
11. De mensen die hebben bijgedragen aan de website begrijpen wat voor mij belangrijk is.					
12. Ik vertrouw de informatie op de website.					
13. Ik zou de website raadplegen als ik een beslissing zou moeten nemen over mijn gezondheid.					
14. Ik heb een gevoel van solidariteit met andere mensen die de website gebruiken.					
15. Ik kan me identificeren met andere mensen die de website gebruiken.					

Voor vragen gerelateerd aan deze vragenlijst, neem alstublieft contact op met: XXXX

Gaat u verder op de volgende pagina

© 2013, Oxford University Innovation Limited. Alle rechten voorbehouden.

De auteurs, prof. dr. Crispin Jenkinson, mw. Laura Kelly en prof. dr. Sue Ziebland, hebben aanspraak gemaakt op hun morele rechten.

	Selecteer het vakje dat op u van toepassing is.				
**Denkend aan de website die u net bekeken heeft, in hoeverre bent u het wel of niet eens met de volgende uitspraken?**	Helemaal mee oneens	Mee oneens	Noch mee eens, noch mee oneens	Mee eens	Helemaal mee eens
16. In zijn geheel, vind ik de website geruststellend.					
17. Ik waardeer het advies dat gegeven wordt op de website.					
18. De website geeft me het vertrouwen dat ik in staat ben om met mijn gezondheid om te gaan.					
19. Ik heb het gevoel veel gemeen te hebben met andere mensen die de website gebruiken.					
20. De website geeft mij het vertrouwen om mijn gezondheidszorgen aan anderen uit te leggen.					
21. De website helpt me om een beter begrip te hebben van mijn persoonlijke gezondheid.					

Voor vragen gerelateerd aan deze vragenlijst, neem alstublieft contact op met: XXXX

Gaat u verder op de volgende pagina

© 2013, Oxford University Innovation Limited. Alle rechten voorbehouden.

De auteurs, prof. dr. Crispin Jenkinson, mw. Laura Kelly en prof. dr. Sue Ziebland, hebben aanspraak gemaakt op hun morele rechten.

	Selecteer het vakje dat op u van toepassing is.				
**Denkend aan de website die u net bekeken heeft, in hoeverre bent u het wel of niet eens met de volgende uitspraken?**	Helemaal mee oneens	Mee oneens	Noch mee eens, noch mee oneens	Mee eens	Helemaal mee eens
22. De website moedigt mij aan om een actievere rol te spelen in mijn gezondheidszorg.					
23. De website geeft mij meer vertrouwen om mijn gezondheid te bespreken met mensen in mijn omgeving (bijvoorbeeld mijn familie of collega's).					
24. Foto's en andere afbeeldingen op de website werden passend gebruikt.					
25. Ik vond de afbeeldingen op de website verontrustend.					
26. De website is gemakkelijk te gebruiken.					

© 2013, Oxford University Innovation Limited. Alle rechten voorbehouden.

De auteurs, prof. dr. Crispin Jenkinson, mw. Laura Kelly en prof. dr. Sue Ziebland, hebben aanspraak gemaakt op hun morele rechten.
