# Supplementary material for: Translation of the eHealth Impact Questionnaire for a Population of Dutch Electronic Health Users: Validation Study
Source: J Med Internet Res. 2019 Aug 26;21(8):e13408. doi: 10.2196/13408 (PMC6732971; doi:10.2196/13408)
Supplement: Multimedia Appendix 2 [file jmir_v21i8e13408_app2.pdf]

Multimedia Appendix 2. Structural Validity: Exploratory Factor Analysis factor loadings.

Item	Factor 1	Factor 2	Factor 3
**A: Study Sample 1 – eHIQ-NL Part 1**			
Part 1 - item 9	0.772*		
Part 1 - item 8	0.663*		
Part 1 - item 11	0.592*		
Part 1 - item 6		0.863*	
Part 1 - item 7		0.759*	
Part1 - item 10		0.612*	
Part 1 - item 4			-0.826*
Part 1 - item 5			-0.670*
Part 1 - item 3			-0.620*
Part 1 - item 1			-0.455*
Part 1 - item 2		0.312*	-0.434*
**B: Study Sample 1 – eHIQ-NL Part 2**			
Part 2 - item 23	0.787*		
Part 2 - item 22	0.676*		
Part 2 - item 7	0.657*		
Part 2 - item 21	0.616*		
Part 2 - item 20	0.592*		
Part 2 - item 18	0.589*		
Part 2 - item 1	0.560*		
Part 2 - item 13	0.401*		
Part 2 - item 9		0.722*	
Part 2 - item 6		0.676*	
Part 2 - item 3		0.541*	
Part 2 - item 5		0.519*	
Part 2 - item 26		0.498*	
Part 2 - item 12		0.481*	
Part 2 - item 24		0.435*	
Part 2 - item 2		0.416*	
Part 2 - item 25		-0.390*	
Part 2 - item 15			0.842*
Part 2 - item 14			0.799*
Part 2 - item 19			0.592*
**C: Study Sample 2 – eHIQ-NL Part 1**			
Part 1 - item 9	-0.453*		
Part 1 - item 11	-0.432	0.351	

Item	Factor 1	Factor 2	Factor 3
Part 1 - item 8	-0.334	0.333	
Part 1 - item 7		0.911*	
Part 1 - item 6		0.791*	
Part 1 - item 10	-0.371	0.658*	
Part 1 - item 4			-0.759*
Part 1 - item 3			-0.736*
Part 1 - item 2		0.310	-0.617**
Part 1 - item 1			-0.596**
Part 1 - item 5			-0.544*
**D: Study Sample 3 – eHIQ-NL Part 2**			
Part 2 - item 22	0.745*		
Part 2 - item 23	0.669*		
Part 2 - item 7	0.517*		
Part 2 - item 1	0.505*	-0.319	
Part 2 - item 8	0.505*		
Part 2 - item 10	0.455*	-0.389	
Part 2 - item 18	0.415*		
Part 2 - item 13	0.355*		
Part 2 - item 21	0.204*		
Part 2 - item 9		-0.849*	
Part 2 - item 6		-0.847*	
Part 2 - item 12		-0.693*	
Part 2 - item 5		-0.631*	
Part 2 - item 17		-0.613*	
Part 2 - item 26		0.583*	
Part 2 - item 2		-0.598*	
Part 2 - item 25		0.583*	
Part 2 - item 11		-0.495*	
Part 2 - item 3		0.447*	
Part 2 - item 4	0.392	-0.431*	
Part 2 - item 24		-0.420*	
Part 2 - item 16		-0.402*	
Part 2 - item 19			0.916*
Part 2 - item 15			0.790*
Part 2 - item 14			0.639*
Part 2 - item 20	0.463*		0.466*

Standardized Factor Loadings. Loadings < .30 suppressed.
