# Supplementary material for: Translation of the eHealth Impact Questionnaire for a Population of Dutch Electronic Health Users: Validation Study
Source: J Med Internet Res. 2019 Aug 26;21(8):e13408. doi: 10.2196/13408 (PMC6732971; doi:10.2196/13408)
Supplement: Multimedia Appendix 3 [file jmir_v21i8e13408_app3.pdf]

Multimedia Appendix 3. Internal Consistency.

Original factor structure		Modified factor structure		Modified factor structure problematic items
**Subscale**	**Omega**	**Subscale**	**Omega**	**Cronbach's Alpha**
**A: Study Sample 1 – eHIQ-NL[1] Part 1**				
**Part 1: General attitude**	0.84	**Part 1: General attitude**	0.90	[2]
Attitudes towards online health information	0.79	Attitudes towards online health information	0.79	
Attitudes towards sharing health experiences online	0.78	Comfort with sharing health experiences online	0.73	
		Usefulness of sharing health experiences online	0.83	
**B: Study Sample 1 – eHIQ-NL Part 2**				
**Part 2: Specific attitude**	0.90	**Part 2: Specific attitude**	0.89	
Confidence and identification	0.85	Motivation and confidence to act	0.85	
Information and presentation	0.70	Information and presentation	0.78	
Understanding and motivation	0.81	Identification	0.82	
**C: Study Sample 2 – eHIQ-NL Part 1**				
**Part 1: General attitude**	0.90	**Part 1: General attitude**	0.91	
Attitudes towards online health information	0.81	Attitudes towards online health information	0.81	
Attitudes towards sharing health experiences online	0.88	Comfort with sharing health experiences online	0.76	
		Usefulness of sharing health experiences online	0.86	
**D: Study Sample 3 – eHIQ-NL Part 2**				
**Part 2: Specific attitude**	0.87	**Part 2: Specific attitude**	0.89	0.91
Confidence and identification	0.92	Motivation and confidence to act	0.85	0.91
Information and presentation	0.65	Information and presentation	0.70	0.91
Understanding and motivation	0.83	Identification	0.86	0.91

1 eHIQ-NL: Dutch version of the e-Health Impact Questionnaire.
2 Cronbach alpha not applicable when omega could be reliably calculated.
